# Investigating the Spatial Heterogeneity of Microbial Communities and Flavor Compounds During the First Fermentation Stage of Sauce-Flavor Baijiu

**DOI:** 10.3390/foods15111849

**Published:** 2026-05-23

**Authors:** Fangling Wen, Feifei Lu, Yu Zhao, Jiehua You, Shengfeng Li, Minna Yao, Xitao Cao, Yonghui Lin

**Affiliations:** 1 Fujian Key Laboratory of Quality and Safety of Agri-Products, Institute of Quality Standards and Testing Technology for Agro-Products, Fujian Academy of Agricultural Sciences, Fuzhou 350003, China; 52309010075@fafu.edu.cn (F.W.); lff860102@163.com (F.L.); 18635315598@163.com (Y.Z.); 2 College of Food Science, Fujian Agriculture and Forestry University, Fuzhou 350002, China; yaominna_fafu@163.com; 3 Fujian Shuanglong Xizhu Baijiu Industry Co., Ltd., Jian’ou 353100, China; 18679330999@163.com (J.Y.); shengfenglee@163.com (S.L.); 4 College of Biotechnology, Jiangsu University of Science and Technology, Zhenjiang 212100, China; xtcao668@just.edu.cn

**Keywords:** sauce-flavor baijiu, fermented grains, spatial heterogeneity, microbial communities, flavor compounds

## Abstract

The first fermentation round serves as the critical starting point for the formation of the sauce-flavor style. However, the spatial heterogeneity and intrinsic relationships between the microbial community, physicochemical properties, and flavor compounds in the first-round fermented grains remain unclear. In this work, microbial communities and volatile compounds across different layers of the fermentation pit were investigated. Results showed both acidity and ethanol content decreased with increasing depth. *Lactobacillus* was the dominant bacterial genus across all layers, whereas the dominant fungal genera exhibited marked spatial stratification: *Trichosporon* prevailed in the bottom layer, while *Pichia* dominated the middle and upper layers. Ethyl phenylacetate served as the backbone aroma compound across all layers, but distinct layer-specific flavor compounds were identified. Nonanal, potentially correlated with *Trichosporon*, was the key flavor compound in the bottom layer; phenolic compounds characterized the middle layer, and *Pichia*-associated esters such as isoamyl acetate dominated the upper layer. Correlation analysis confirmed significant associations between specific microbial taxa and ester accumulation. Notably, *Pichia* and *Trichosporon* exhibited opposing correlations with multiple flavor compounds, suggesting metabolic differentiation. This study clarified the spatial distribution of microorganisms and flavors and provided correlational insights into flavor formation in traditional baijiu fermentation.

## 1. Introduction

Sauce-flavor baijiu is produced from sorghum, wheat, and water using traditional solid-state fermentation techniques and the ancient “12987” method, which involves nine rounds of steaming, eight cycles of fermentation, and seven rounds of distillation [1,2]. Its unique sauce aroma originates entirely from complex microbial fermentation processes [3]. “Fermented grains (FG)”, the core matrix of solid-state fermentation, constitute a mixture of raw materials partially degraded by microorganisms, rich in residual grains, water, and fermentation metabolites [4]. FG provides the essential nutrients and microenvironment for brewing microorganisms and harbors a complex ecosystem comprising bacteria (including lactic acid bacteria and caproic acid bacteria), fungi (including yeasts), and methanogenic archaea, among others [5,6]. The flavor profile of FG is a direct manifestation of the metabolic activities of its internal microbial community and serves as the foundation for the quality of the base liquor. Therefore, elucidating the relationship between microorganisms and flavor compounds in FG is crucial for enhancing baijiu quality and advancing intelligent brewing.

The dynamic succession and spatial heterogeneity of the microbial community have directly driven flavor formation throughout the brewing cycle [7]. Studies confirmed that *Lactobacillus* was the overwhelmingly dominant genus in the bacterial community of FG across multiple fermentation rounds. Its relative abundance had gradually decreased with advancing rounds, and significant spatial heterogeneity existed in microbial communities among the upper, middle, and lower layers of FG, leading to variations in metabolic function [8,9,10]. Metatranscriptomic analysis revealed that microorganisms in FG from different spatial positions exhibited significant differences in metabolic pathways such as RNA degradation and glycolysis, with the microbial community in the lower layer making a particularly prominent contribution to overall metabolism [10]. However, existing research on the correlation between microbes and flavor had been highly concentrated on rounds 3 to 5 (the peak production period), systematically revealing the relationship between stable microbial communities and the main flavor metabolic network during this stage [10,11]. In contrast, the first-round FG, the starting point of the fermentation cycle, during which the microbial community was established and key flavor precursors such as lactic acid and acetic acid began to accumulate [12], received little attention. A systematic analysis of the microbe–flavor correlation at this source stage will prevent a fundamental understanding of the initial construction logic of brewing flavor.

Targeting the first-round pit fermentation system of sauce-flavor baijiu from a distillery at Jian’ou in Fujian Province, this study integrated multi-omics technologies, including high-throughput sequencing and headspace solid-phase microextraction coupled with gas chromatography–mass spectrometry (HS-SPME-GC–MS). Combined with multivariate statistical analysis, it systematically elucidated the interactions among the physicochemical properties of FG, the dynamics of the microbial community, and the flavor compound profile. A quantitative correlation network between the microbial flora and characteristic flavor compounds under spatial heterogeneity during the first fermentation round was constructed, aiming to provide a theoretical basis and data support for the directional regulation of the solid-state fermentation process.

## 2. Materials and Methods

### 2.1. Materials and Reagents

FastPure Stool DNA Isolation Kit was obtained from TransGen Biotechnology Co., Ltd., Beijing, China. Agarose (Cat. No. 72210019) was purchased from Thermo Fisher Scientific, MA, USA. Methanol (LC MS grade) and acetonitrile (MS grade) were obtained from Thermo Fisher Scientific Co., Ltd., Shanghai, China. 2-Octanol (standard) was purchased from Beijing Solarbio Science & Technology Co., Ltd., Beijing, China. Soluble starch, sodium hydroxide, potassium hydrogen phthalate, phenolphthalein, bromocresol green, and methyl red were obtained from Xilong Scientific Co., Ltd., Shanghai, China. Sulfuric acid was purchased from Hengyang Kaixin Chemical Reagent Co., Ltd., Hengyang, China. Anhydrous sodium carbonate was obtained from Tianjin Chemical Reagent Research Institute Co., Ltd., Tianjin, China. All other reagents were of analytical grade. Ultrapure water was prepared using a water purification system (WP 2RO WF 10S, Watpur, Chengdu, China).

### 2.2. Instruments and Equipment

Volatile Compounds in FG were analyzed using a gas chromatograph–mass spectrometer (GCMS QP2020 NX, Shimadzu, Kyoto, Japan). High-throughput sequencing was conducted by the Illumina MiSeq (Illumina, San Diego, CA, USA) platform. DNA amplification was performed using the ABI GeneAmp^®^ 9700 PCR thermal cycler, Shanghai, China; quantification of the amplified DNA was carried out with the blue fluorescence quantitative system (QuantiFluor™ ST, Shanghai, China).

### 2.3. Methods

#### 2.3.1. Sample Collection

The 1st round fermented grains (FGs) during the pit fermentation were sampled on 17 December 2024, at a baijiu distillery in Jian’ou City, Fujian Province, China. As shown in Figure S1, four corners and the center of FGs from the upper layer (0.3–0.6 m), middle layer (1.9–2.1 m), and bottom layer (3.5–3.7 m) selected were collected, respectively, and mixed completely. The collected samples were named FGU, FGM, and FGB, respectively. All FG samples were sealed in sterile bags, transported under cold conditions, and stored at −80 °C for subsequent microbiological analyses. Each layer sample has three biological replicates, totaling nine samples.

#### 2.3.2. Determination of Physicochemical Indices of FG

Alcohol content, total acidity, moisture, and starch in FG were determined according to the standard [13]. Reagent preparation followed these standards [14,15]. Alcohol content, total acidity, moisture and starch content were measured by distillation alcoholmeter method, acid base titration, oven-drying method, and acid hydrolysis glucose determination method, respectively.

#### 2.3.3. Total DNA Extraction, PCR Amplification, and High-Throughput Sequencing of FG

Metagenomic DNA was extracted from FG samples according to the instructions of the MagAttract PowerSoil Pro DNA Kit (Qiagen, Hilden, Germany). PCR technology was used to amplify target microbial DNA for high-throughput sequencing analysis. The V3 V4 region of bacterial 16S rRNA genes was amplified with universal primers 338F (5′ACTCCTACGGGAGGCAGCAG 3′) and 806R (5′GGACTACHVGGGTWTCTAAT 3′). The ITS1 ITS2 region of fungal rRNA genes was amplified with primers ITS1F (5′CTTGGTCATTTAGAGGAAGTAA 3′) and ITS2R (5′GCTGCGTTCTTCATCGATGC 3′). PCR conditions are shown in detail in Supplementary Materials Section S1.1. High-throughput sequencing was carried out followed the steps in Supplementary Materials Section S1.2.

#### 2.3.4. Determination of Volatile Compounds in FG

The analysis of FG samples was carried out following the method described by Ref. [16]. Precisely 2.0 g of the FG sample was added into a 20 mL headspace vial, followed by the addition of 5 mL of saturated NaCl solution and 20 μL of 2-octanol (0.9 mg/mL) as the internal standard. The mixture was sonicated at 100 W for 10 min. Headspace solid-phase microextraction (HS-SPME) was performed using an Rtx-5MS column (Shimadzu, Kyoto, Japan)(30.0 m length × 0.25 mm i.d. × 0.25 μm film thickness). The sample was preincubated at 50 °C for 10 min, followed by extraction for 30 min, after which it was directly injected. Desorption was carried out at 250 °C for 5 min. The SPME fiber was conditioned at 260 °C for 20 min between runs. The initial oven temperature was 40 °C, held for 3 min, increased at 2 °C·min^−1^ to 150 °C (holding for 2 min), and further increased at 6 °C·min^−1^ to 230 °C (holding for 15 min). High-purity helium (99.999%) carrier gas flow was maintained at 1.5 mL·min^−1^. Electron ionization (EI) was at 70 eV, ion source temperature was 230 °C, and a scan range of m/z was 35~350.

Volatile compounds were identified by matching against the NIST database (match factor > 85%) and relatively quantified using the internal standard method based on peak area ratios. The formula calculating the concentration and relative odor activity value (ROAV) can be seen in Supplementary Materials Section S1.3 following the approach described by Lin [17].

### 2.4. Data Processing

Data were presented as mean ± standard deviation. Statistical processing and Spearman correlation analysis were performed using SPSS 22.0. Differences among groups were assessed by one-way ANOVA, with statistical significance set at *p* < 0.05. Graphs were generated with GraphPad Prism (v 11.0.0) software. Correlation heatmaps were plotted to visualize associations between volatile compounds and microbial taxa.

## 3. Results

### 3.1. Changes in Physicochemical Parameters of Fermented Grains During the First Fermentation Cycle

**Figure 1:**
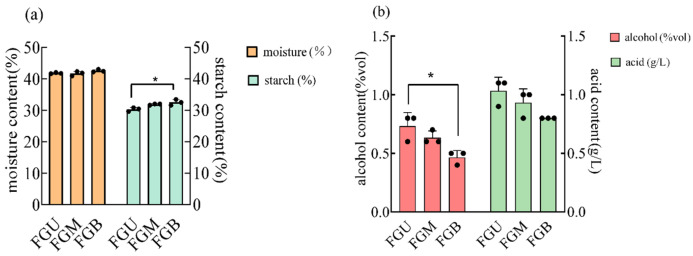
Changes in the physicochemical parameters (moisture, starch, alcohol content, acidity) of fermented grains across different layers in the fermentation pit. Note: FGU, FGM, and FGB correspond to the fermented grain samples from the upper, middle, and bottom layers of the pit-fermented grains, respectively. (**a**) Changes in moisture and starch content across different layers of fermented grains; (**b**) Changes in alcohol content and total acidity across different layers of fermented grains; * *p* < 0.05 indicates statistical significance.

As shown in Figure 1, ethanol concentration and total acidity decreased with increasing depth. Specifically, acidity decreased from 1.1 g/L in the upper layer to 0.8 g/L in the bottom layer, while ethanol content declined from 0.7% vol to 0.4% vol. This might be related to the environmental gradients within the fermented grains and the influence of microbial differences among layers [18]. However, there was no significant difference in moisture content among the three layers, while starch content differed between FGU and FGB. During the fermentation process of Jiangxiang-style baijiu in pits, the microenvironments varied at different depths. The upper layer generally experienced lower average temperatures, while the lower layer had relatively higher temperatures. Additionally, oxygen accessibility might also change with depth [19,20]. These layered environmental differences could potentially correlate with the distribution and metabolic profiles of bacterial and fungal communities, further possibly relating to the variation in organic acid and ethanol accumulation. It should be noted that this study has limitations: real-time monitoring of in situ environmental parameters (pH, temperature, dissolved oxygen) and metabolic indicators (organic acids, fermentable sugars) was not performed.

*Lactobacillus* was the dominant microbial group and the primary producer of organic acids in the fermented grains across all layers during the first fermentation round. *Pichia* yeasts were Pasteur-positive, and their metabolism was strictly regulated by oxygen: the microaerophilic upper layer supports respiratory growth (relative abundance 61.4%) and fermentative ester production, whereas the strictly anaerobic bottom layer inactivated their respiratory chain, allowing anaerobically adapted *Trichosporon* to compete with them. These physiological characteristics collectively were consistent with the progressive vertical decline in acidity and ethanol content [21,22].

In this round, the dominant microbial community directed metabolism toward acid production rather than ethanol production. High-throughput sequencing results confirmed that *Lactobacillus* was the absolutely dominant bacterial genus across all layers, and its primary metabolic product was lactic acid rather than ethanol [23]. Furthermore, the dominant fungi identified in this study were *Pichia* (JCU, JCM) and *Trichosporon* (JCB), both of which were non-*Saccharomyces* yeasts whose major metabolic functions in baijiu fermentation involved ester synthesis and substrate decomposition, rather than high ethanol yield [24]. The fact that high-ethanol-yielding *Saccharomyces cerevisiae* had not yet become the dominant microbial community in the first round may be one of the factors associated with the relatively low ethanol accumulation. Finally, the high-temperature fermentation characteristic of sauce-flavor baijiu accelerated the proliferation of *Lactobacillus* and the accumulation of organic acids, while simultaneously suppressing yeast-mediated ethanol generation, thereby fundamentally limiting the ethanol yield in the first-round fermentation [25,26]. The physicochemical gradients observed in the present study, with acidity decreasing from 1.1 g/L (FGU) to 0.8 g/L (FGB) and ethanol from 0.7% vol to 0.4% vol (Figure 1), supported the characterization of the first fermentation round as both the “foundation period” for microbial community establishment based on starter inoculation and the “primary accumulation period” for key flavor precursors such as lactic acid and acetic acid [12]. The dominance of *Lactobacillus* and the spatial stratification of ester-producing yeasts revealed in this study suggested that the microbial composition and activity established during this stage may influence the potential and direction of subsequent flavor evolution during later peak-production rounds.

### 3.2. Microbial Community Diversity During the First Fermentation Cycle in Baijiu Pits

#### 3.2.1. Diversity Analysis of Bacterial and Fungal Communities in Fermented Grains

Alpha-diversity analysis was performed on fermented grain (FG) samples to assess the richness and diversity of microbial communities. ACE, Chao, Shannon, and Simpson indices were calculated to characterize the bacterial and fungal community structures in different layers (JCU, JCM, JCB) of fermented grains in the pit. Detailed calculation formulas can be found in the Supplementary Materials Section S1.4. The results (Table 1) indicated that the bacterial richness (Chao index) and diversity (Shannon index) in the JCB layer were significantly higher than those in the JCU and JCM layers. In contrast, the richness and diversity of fungi were significantly higher in the JCU and JCM layers than in the JCB layer. The Coverage index for all samples exceeded 0.999, demonstrating a sufficiently high sequencing depth to detect the vast majority of microbial sequences in the samples. This ensured that the results reliably reflected the true composition of bacterial and fungal communities in the fermented grains.

**Figure 2:**
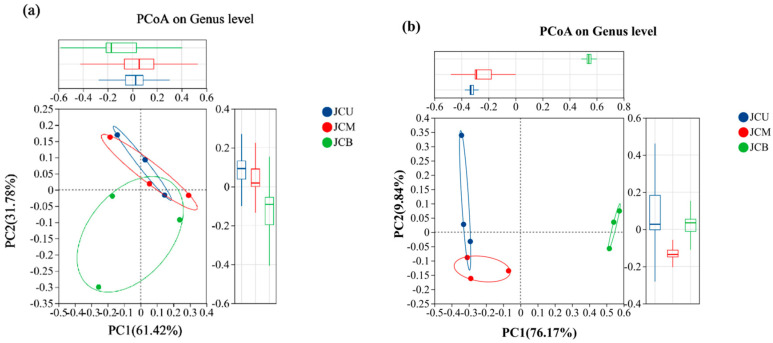
Principal coordinate analysis of bacteria and fungi in different layers of cellar mash based on genus level. Note: (**a**) PCoA plot at the bacterial genus level; (**b**) PCoA plot at the fungal genus level. Points in different colors or shapes represent samples from different groups. A closer distance between two sample points indicates greater similarity in their genera composition.

As shown in Figure 2, PCoA analysis at the genus level revealed differences in the microbial communities of the fermented grains. For bacterial communities (Figure 2a), the JCU and JCM groups showed similar distributions along the PC1 axis (explained variance: 61.42%), whereas the JCB group exhibited a tendency to separate along the PC2 axis (explained variance: 31.78%). For fungal communities (Figure 2b), the JCU and JCM groups clustered closely along the PC1 axis (explained variance: 76.17%) and were separated from the JCB group along the PC2 axis (explained variance: 9.84%). Combined with ANOSIM analysis (bacteria: R^2^ = 0.05, *P* = 0.7; fungi: R^2^ = 0.7, *P* = 0.001) and the aforementioned alpha diversity indices, the results indicated that the bacterial community in the JCB layer differed from those in the other layers, while the fungal communities in the JCU and JCM layers shared similar structures and exhibited relatively higher diversity compared to the JCB layer.

#### 3.2.2. Analysis of Bacterial and Fungal Community Composition and Genera Divergence in Fermented Grains

**Figure 3:**
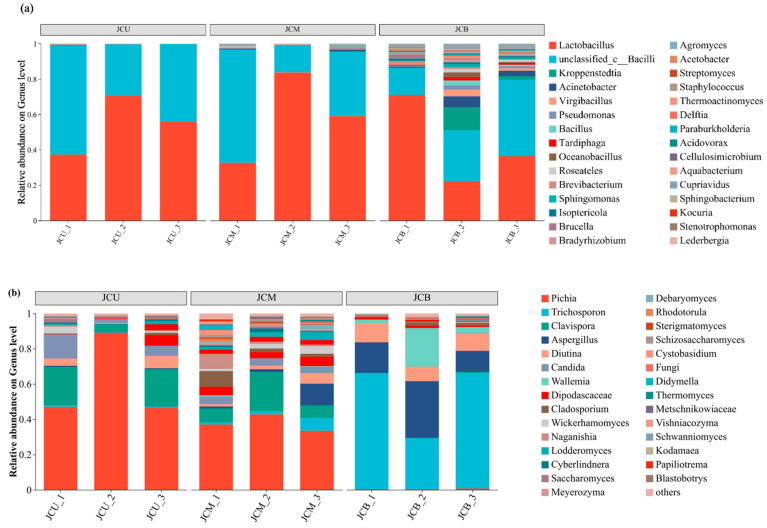
Stacked bar charts showing the relative abundance of bacterial and fungal genera in fermented grain samples from different layers of the fermentation pit. Note: (**a**) stacked bar chart of the relative abundance of bacterial genera; (**b**) stacked bar chart of the relative abundance of fungal genera. The x-axis represents the sample names, and the y-axis indicates the proportion of genera within each sample. Columns of different colors represent different genera, and the length of each column corresponds to the relative proportion of that genus.

Microorganisms play a crucial role in the brewing process of baijiu [27]. Analysis of bacterial community composition at the genus level (as shown in the bar chart) identified a total of 226 bacterial genera in the fermented grains from three different layers: JCU, JCM, and JCB. It was particularly noted that all occurrences of the genus name *Lactobacillus* in this paper referred to the name after the taxonomic change. Among these, *Lactobacillus* was the overall dominant genus, which was consistent with the findings reported by Wei [28] for the first fermentation cycle of Jiangxiang-style baijiu, where this genus served as the predominant bacterial group across all fermentation layers. Similarly, Liang [29] also noted that *Lactobacillus* was the dominant bacterial genus during the fermentation process of sauce-flavor baijiu. In Figure 3a, *Lactobacillus* is the dominant microbial group. Studies have shown that many *Lactobacillus* strains isolated from baijiu fermentation environments were capable of decomposing starch and fermenting it to produce lactic acid [30]. The accumulation of organic acids and the subsequent increase in H^+^ concentration led to a decrease in the pH of the fermented grains and a corresponding increase in acidity. In addition to *Lactobacillus*, other bacterial genera were detected in each layer, though their abundances were generally low. For example, *unclassified_c_Bacilli*, *Kroppenstedtia*, and *Acinetobacter* were present in the bottom layer (JCB) but accounted for only minor proportions. Notably, the bacterial composition in the JCB layer differed significantly from that in the JCM and JCU layers, with genera such as *Kroppenstedtia*, *Acinetobacter*, *Virgibacillus*, *Pseudomonas*, *Bacillus*, and *Oceanobacillus* being either unique to or relatively enriched in JCB. This further reflected the influence of vertical spatial heterogeneity within the fermentation pit on bacterial community structure.

In terms of fungi, genus-level composition analysis identified a total of 118 fungal genera in the fermented grains from the first fermentation cycle. As shown in Figure 3b, *Pichia* was the dominant fungal genus in the middle and upper layers (JCU and JCM), with relative abundances of 61.4% and 37.4%, respectively. In contrast, the bottom layer (JCB) was dominated by *Trichosporon*, with an abundance of 32.1%. Additionally, *Clavispora* and *Aspergillus* also showed relatively high abundances in the middle and upper layers. Other fungal genera, such as *Diutina*, were distributed across all layers but exhibited low relative abundances.

**Figure 4:**
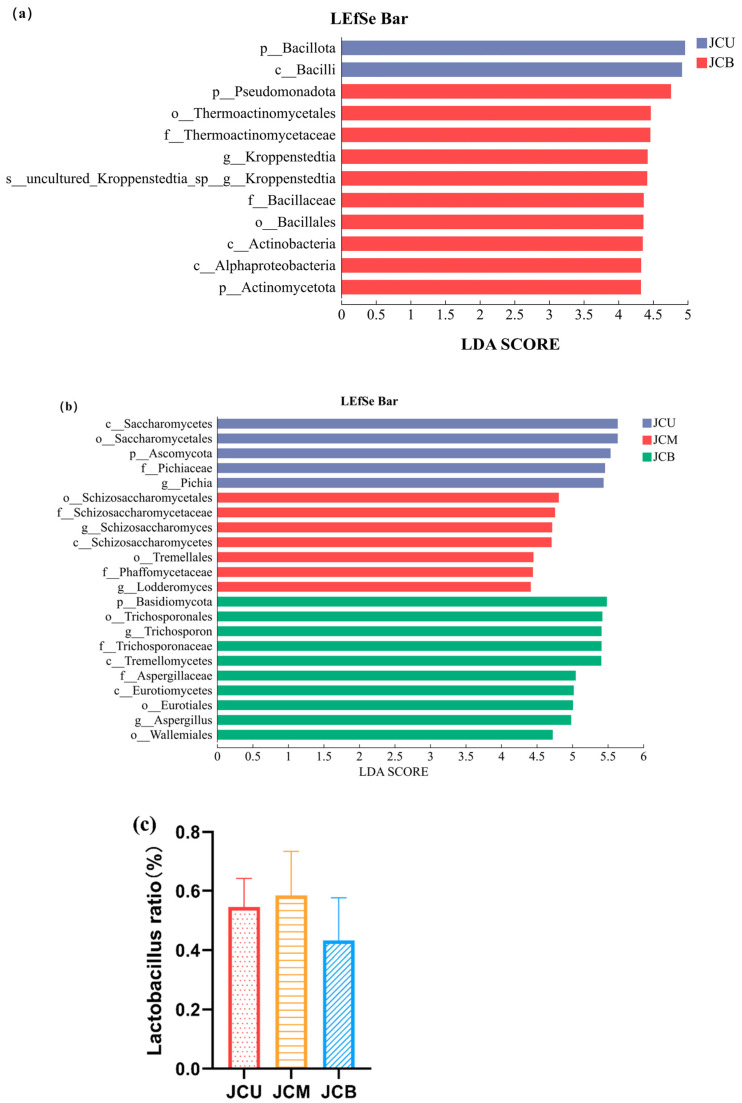
LEfSe analysis of bacterial and fungal genera in fermented grain samples from different layers of the fermentation pit. Note: (**a**) LEfSe-based bar plot showing differential analysis at the bacterial genus level; (**b**) LEfSe-based bar plot showing differential analysis at the fungal genus level; (**c**) bar chart showing the relative abundance of the genus *Lactobacillus* across different fermented grain layers. The LDA (linear discriminant analysis) bar chart illustrates microbial taxa that play significant roles across multiple groups. The LDA scores, derived from LDA analysis, reflect the extent to which genera abundance contributes to the observed differences: a higher LDA score indicates a greater influence of the genera abundance on the group distinction.

LEfSe analysis (LDA > 4.0, *p* < 0.05) of the bacterial communities across different layers of the pit-fermented grains identified a total of 13 bacterial taxa with significantly differential abundances (seen in Figure 4a). The results indicated that each fermented grain layer harbored its own signature bacterial taxa: the upper layer (JCU) was characterized by the phylum *Bacillota* and the class *Bacilli* within it, whereas the bottom layer (JCB) was primarily distinguished by taxa such as the phylum *Pseudomonadota* and the order *Thermoactinomycetales*. Regarding the fungal communities, in Figure 4b, LEfSe analysis (LDA > 4.0, *p* < 0.05) identified 25 fungal taxa exhibiting significant abundance differences. The results revealed a clear spatial stratification in the fungal community as well: the upper layer (JCU) was characterized by the class *Saccharomycetes* and related genera such as *Pichia*; the middle layer (JCM) featured significant taxa including the families *Schizosaccharomycetaceae* and *Phaffomycetaceae* and the genus *Lodderomyces*; the bottom layer (JCB) was predominantly composed of taxa such as the phylum *Basidiomycota*, the order *Trichosporonales*, and the genus *Aspergillus.* Furthermore, as shown in Figure 4c, the genus *Lactobacillus* was detected across all fermented grain layers, but its relative abundance varied among the different strata. This further supported the notion that the gradient changes in the fermentation microenvironment exerted a selective influence on the spatial distribution of specific functional microbial groups. The clear stratification of microbial communities observed across pit layers (Figure 3 and Figure 4) was understood in terms of the defining traits of core functional microorganisms, which typically exhibited direct synthesis of key flavor compounds, strong environmental adaptability, and dominance at specific fermentation stages [31,32]. In this study, *Lactobacillus* exemplified these traits: it dominated all layers (relative abundance >90%), consistent with the established pattern in which *Lactobacillus* proliferated rapidly during the stacking stage due to the increasing acidity of the grains, and profoundly shaped the subsequent community structure [33].

#### 3.2.3. Correlation Analysis and Functional Prediction of Microorganisms and Physicochemical Factors in Different Layers of Fermented Grains

**Figure 5:**
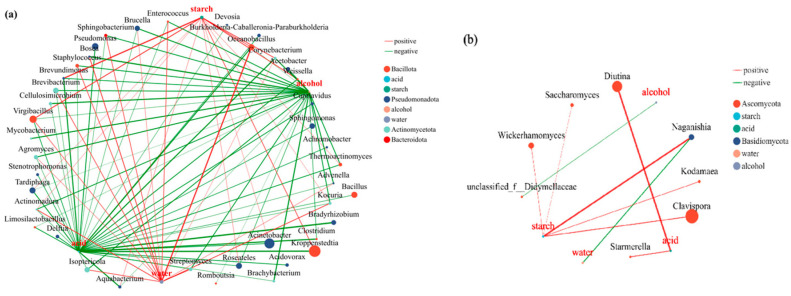
Correlation network heatmap between bacterial/fungal genera and physicochemical factors across different layers of fermented grains in the pit. Note: (**a**) correlation network heatmap between the bacterial community and physicochemical factors; (**b**) correlation network heatmap between the fungal community and physicochemical factors. Spearman’s rank correlation coefficients were calculated between physicochemical factors and taxa at the genus level based on the magnitude of physicochemical data and genera abundance, reflecting the correlations between environmental factors and genera. The network diagram displays only taxa with an absolute correlation coefficient ≥ 0.6 and a significance of *p* < 0.05. Connections in red indicate positive correlations, while green indicates negative correlations. Thicker lines represent stronger correlations between taxa, and a greater number of connections indicates closer associations of a given taxon with others.

As shown in Figure 5a, the correlation network between the bacterial community and physicochemical factors consisted of 45 nodes and 92 edges. Correlation analysis of bacterial genera and physicochemical indicators in the fermented grains of the pit revealed that the bacterial community was primarily negatively correlated with acidity and ethanol content: a total of 37 genera showed a negative correlation with acidity, and 27 genera were negatively correlated with ethanol content. In contrast, bacteria were mainly positively correlated with starch and moisture: starch was positively correlated with 13 genera, with the strongest correlation observed with the genus *Brevundimonas*; moisture was positively correlated with 15 genera, with the highest correlation found with the genus *Oceanobacillus*. As shown in Figure 5b, the correlation network between the fungal community and physicochemical factors comprised 12 nodes and 9 edges. Correlation analysis indicated that the acidity of the fermented grains was positively correlated with the genera *Diutina* and *Starmerella*, with a stronger correlation observed with the former, and research has found that the fungal genus Diutina has a fermentation effect on ethanol production [34]. Ethanol content, on the other hand, was negatively correlated with fungi from the *unclassified-Didymellaceae* family. Starch content was positively correlated with multiple fungal genera, including *Naganishia*, *Clavispora*, *Kodamaea*, *Wickerhamomyces*, and *Saccharomyces*, these findings were similar to the research results of Ref. [35].

**Figure 6:**
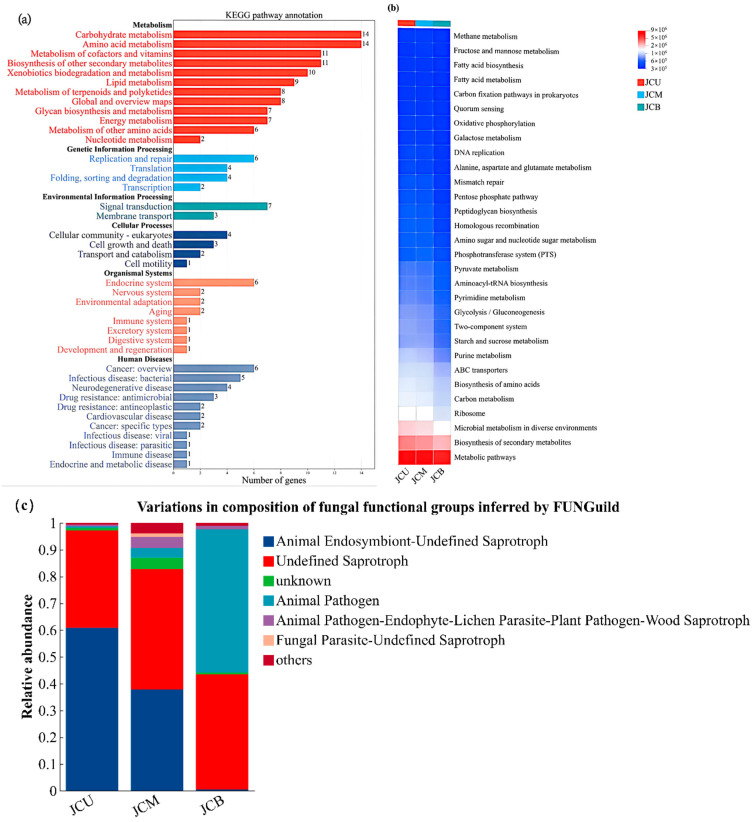
Functional guild prediction chart of bacteria and fungi. Note: (**a**) KEGG pathway annotation of bacteria in the fermented grains; (**b**) heatmap of KEGG functional abundance in the fermented grains; (**c**) variation in fungal functional guild composition inferred using FUNGuild.

As shown in Figure 6, the bacterial functional profiles were predicted using PICRUSt with reference to the KEGG database. The results clearly illustrated the functional potential of the bacterial communities across different fermented grain layers. In Figure 6a, a total of six major biological pathways were involved, including metabolism and its subcategories, encompassing 41 secondary functional pathways such as biosynthesis of secondary metabolites, microbial metabolism in diverse environments, carbon metabolism, and starch and sucrose metabolism, reflecting a high degree of functional diversity. In Figure 6b, within the primary functional categories, metabolism accounted for over 50%, indicating its dominant role during the fermentation of the fermented grains. At the secondary functional level, carbohydrate metabolism and amino acid metabolism were the most prevalent, suggesting that the core functions of the microbial community were centered on energy acquisition (carbon utilization) and nutrient synthesis (amino acid metabolism). The microbial community in the fermented grains drove the fermentation process through a core network of carbon metabolism–amino acid metabolism–energy conversion. This functional profile might help generate hypotheses about flavor formation during baijiu fermentation. The observed vertical stratification of fungal functional guilds across different layers (Figure 6c) likely reflected the physicochemical gradients distributed within the fermented grains. The relatively oxygen-rich upper layer might favor the growth of broad-spectrum decomposers. Some taxa predicted as “Animal Endosymbiont-Undefined Saprotrophs” might act as initial colonizers, utilizing their broad enzymatic repertoire for early substrate breakdown, which has been reported in various fermentation ecosystems [36]. The relatively stable presence of “Undefined Saprotrophs” across all layers indicated a core community driving the fundamental decomposition of plant material. This is aligned with findings that saprotrophic fungi served as essential decomposers of organic matter in solid-state fermentations [37]. The annotation of basal fungi as “animal pathogens” did not imply an actual pathogenic risk in the food matrix of baijiu fermentation; their in situ function was more likely to participate in organic matter decomposition and flavor precursor transformation through a saprotrophic nutritional mode, and confirmation required metatranscriptomics or cultivable functional validation [38,39,40]. Their metabolic activities in this unique niche might contribute to flavor formation, although their specific roles required further investigation [41]. These functional predictions indicated that while the core metabolic framework (carbon and amino acid metabolism) was conserved across layers, the spatial differentiation of flavor profiles was likely driven by the variable metabolic contributions of specific fungal and minor bacterial taxa, as explored in the correlation analyses below.

### 3.3. Volatile Compounds in Fermented Grains of the First Round of Pit Fermentation During Liquor Brewing

A total of 44 volatile compounds were identified in the first-round fermented grain samples, comprising 28 esters, 8 alkanes, 3 alcohols, 2 phenols, and 3 other compounds. The relative contents of these volatile compounds in the fermented grains are presented in Table S1. Esters exhibited the highest proportion, followed by alkanes and alcohols. The total relative content of esters remained at a high level throughout the fermentation period. In this study, isoamyl alcohol showed the highest concentration (608.20 ± 9.43 μg/kg), while the content of ethanol was relatively low (3.93 ± 0.11 μg/kg). Higher alcohols played a crucial role in shaping the flavor profile of baijiu, contributing to its fullness, richness, and overall sensory complexity [42,43]. Esters, notable for their diverse varieties and high abundance, significantly contributed to the overall aroma of baijiu. They represented a key distinction between Chinese baijiu and other distilled spirits, particularly in Jiangxiang (sauce-aroma) baijiu. Among the esters, isobutyl lactate was the most abundant. Its concentration was highest in FGM (181.46 ± 5.02 μg/kg) and relatively lower in FGU (107.11 ± 4.94 μg/kg) and FGB (87.18 ± 1.58 μg/kg), which might be attributed to environmental factors and microbial community composition during fermentation. The content of ethyl acetate was higher than that of propyl acetate, with both showing a decreasing gradient from FGU to FGB. In contrast, the concentration of ethyl palmitate was lower in FGM than in FGU and FGB. However, it should be noted that sauce-flavored baijiu underwent multiple rounds of fermentation and distillation, so the volatile compounds detected in the first round represented only an early stage of a complex process, rather than the final aroma profile of the baijiu product.

Based on literature sources, the odor thresholds of 44 aroma compounds were obtained, and their relative odor activity values (ROAVs) were calculated according to the formula described in Supplementary Materials Section S1.3. The aroma contributions of volatile components in the upper, middle, and lower layers of the fermented grains were evaluated, and the results are presented in Table S2. The analysis indicated that the aroma composition of the fermented grains showed certain variations along the vertical spatial gradient, which might reflect the influence of microenvironmental gradients on microbial metabolism and flavor formation during solid-state fermentation. Ethyl phenylacetate (A8) was identified as the benchmark key aroma compound (ROAV = 100) in all three layers, and its relatively high contribution likely forms the foundation of the liquor’s aroma profile [44]. Distinct characteristic aroma compounds were observed in different layers: in the upper layer, isoamyl acetate (A10, ROAV = 25.98) and 4-hydroxy-3-methyl-2-butanone (A14, ROAV = 69.57) showed notable contributions. These compounds were generally associated with yeast metabolic activity, and their distribution might indicate that the relatively oxygen-rich microenvironment in the upper layer favors metabolic pathways such as ester synthesis [45]. In the middle layer, phenolic compounds (e.g., A20 and A37, which were detected only in this layer) exhibited relatively high ROAV. These components may be correlated with microbial transformation or degradation of precursors, though this remains speculative based on correlation data alone; this correlation might suggest that the microbial community or metabolic environment in the middle layer could differ from those in the upper and lower layers. In the lower layer, nonanal (A26) showed a comparatively high ROAV (107.53), a finding that warranted attention. Its aroma characteristics might arise from specific anaerobic metabolic processes or oxidation of unsaturated fatty acids, resulting in a flavor tendency distinct from that of the upper and middle layers [46]. Furthermore, most long-chain fatty acid ethyl esters and alkanes generally had ROAVs below 0.1, indicating their weak contribution to direct aroma perception; their primary role likely lied in influencing the mouthfeel and structure of the liquor. Ethanol (A28) had an ROAV of zero, which was consistent with its role as a solvent rather than a primary aroma-active compound. Overall, the vertical distribution characteristics of flavor in the fermented grains might correspond to gradients in oxygen, temperature, and microbial communities within the fermentation pit. This understanding contributed to a deeper comprehension of the complexity of solid-state fermentation systems and provided a reference for optimizing product flavor profiles through layer-specific management. Therefore, the present findings could be considered as a preliminary contribution towards understanding the aroma formation mechanism.

### 3.4. Analysis of Microbial Community and Physicochemical Composition in Different Layers of the First-Round Pit Fermentation of Baijiu and Correlation Prediction of Their Impact on Volatile Compounds

**Figure 7:**
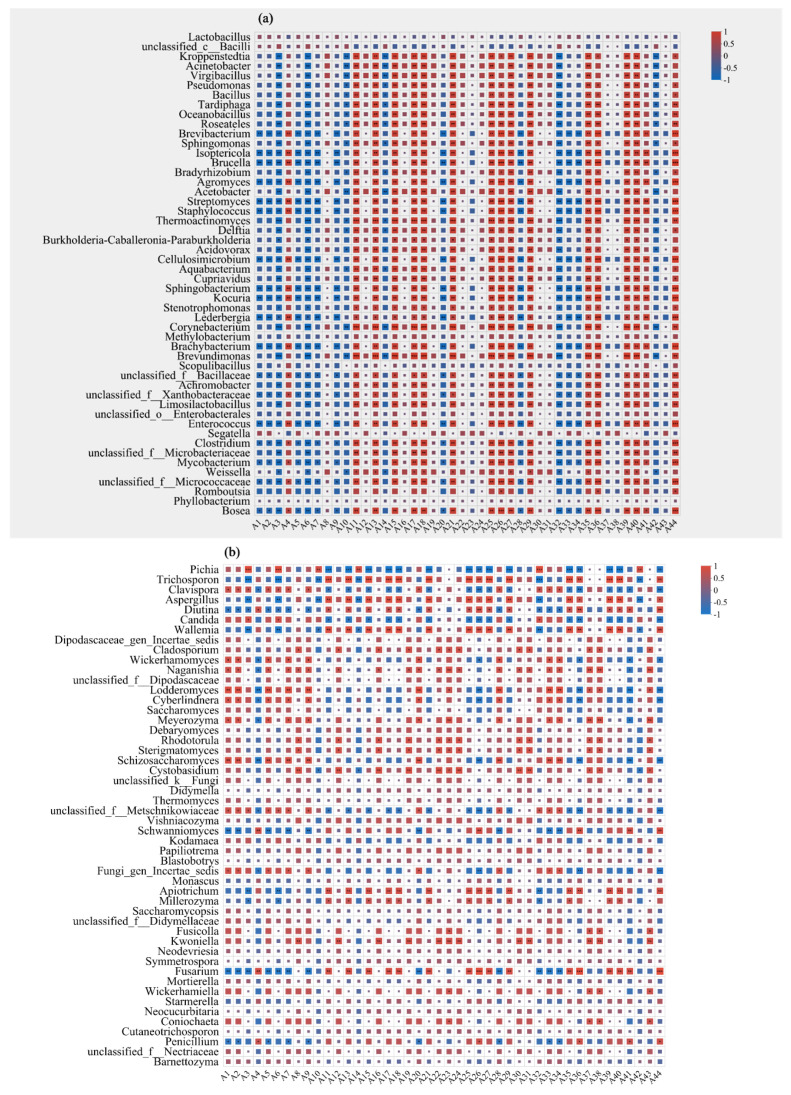
Correlation between microbial community and volatile substances in FG of Fujian Maotai-flavor baijiu during the first round of cellaring. Note: red indicates positive correlation, and blue indicates negative correlation. (**a**) Correlation between bacterial communities and volatile substances; (**b**) correlation between fungal communities and volatile substances. * Indicates significant differences (*: *p* < 0.05; **: *p* < 0.01; ***: *p* < 0.001).

This study employed Spearman’s correlation analysis, focusing on the top 50 microbial genera by relative abundance, to explore the potential associations between bacterial and fungal communities and volatile flavor compounds in the fermented grains. In the analysis of the bacterial community at the genus level (Figure 7a), a heatmap showed certain positive and negative correlations between some microbial genera and specific flavor substances. For instance, *Korppenstedtia* showed significant positive correlations with ethyl elaidate (A11), ethyl octanoate (A13), ethyl phenylacetate (A17), and ethyl laurate (A18) (*p* < 0.01), while exhibiting significant negative correlations with ethyl acetate (A3), propyl acetate (A6), and isoamyl acetate (A10) (*p* < 0.01). Similarly, *Virgibacillus* showed significant positive correlations with ethyl phenylacetate (A17), ethyl laurate (A18), ethyl decanoate (A21), and ethyl nonanoate (A27). Its spectrum of negatively correlated compounds highly overlapped with that of *Korppenstedtia*, also including ethyl acetate (A3), propyl acetate (A6), and isoamyl acetate (A10). These results suggested the possible existence of specific functional groups within the bacterial community, whose metabolic orientation might be associated with the accumulation trends of particular esters.

In the correlation analysis of key fungal communities (Figure 7b), more complex association patterns were observed. *Pichia* showed positive correlations with ethyl acetate (A3), propyl acetate (A6), and ethyl stearate (A32) but negative correlations with compounds such as ethyl elaidate (A11), ethyl octanoate (A13), 1-pentadecene (A15), ethyl phenylacetate (A17), and ethyl laurate (A18). Conversely, *Trichosporon* showed positive correlations with the latter group of compounds and negative correlations with the former group. The observation that *Pichia* and *Trichosporon* exhibited opposite correlation trends with some flavor compounds provided potential clues for understanding microbial community succession during fermentation. As shown in Figure 3, the relative abundance of the dominant genus *Pichia* increased gradually, while that of *Trichosporon* decreased during fermentation. Their opposing association spectra with flavor compounds could be interpreted as differentiation or competition in their metabolic functions within the fermentation niche. Nonanal was the key flavor compound characterizing the bottom layer, and its accumulation showed a statistically significant correlation with the relative abundance of *Trichosporon* (Figure 7b). Although direct genomic evidence for an unsaturated fatty acid oxidation pathway in this genus was beyond the scope of amplicon-based sequencing, the literature indicated that *Trichosporon* genera possessed lipolytic capabilities that might facilitate the release of linoleic acid, the precursor of nonanal [47,48,49]. Verification of the actual metabolic pathway would require metagenomic or metatranscriptomic analysis in future studies.

In light of the existing literature, the key flavor esters in baijiu were diverse, and their formation was closely related to microbial activity [50]. The positive correlations observed in this study between *Pichia* and ethyl acetate/propyl acetate were consistent with previous conclusions identifying *Pichia* yeast as an important ester-producing microorganism. Studies have shown that *Pichia* could activate key enzymes and promote glucose uptake under specific conditions, possibly contributing to the accumulation of metabolites like ethyl acetate. Solid-state fermentation of baijiu was a complex process involving multiple microorganisms. Differences in microbial composition could form distinct micro-ecosystems, potentially leading to variations in the synthesis pathways and accumulation levels of flavor substances. This correlation analysis suggested potential associations between specific microbial genera and the accumulation of a series of ester compounds, offering a reference for exploring the coupling mechanism between microbial functional succession and flavor formation.

## 4. Conclusions

This study systematically revealed that the baijiu fermentation pit constituted a highly differentiated micro-ecosystem along a vertical gradient for the first time. The microbial communities within the fermented grains, such as *Pichia* in the upper layer and *Trichosporon* in the bottom layer, exhibited clear spatial stratification. Their abundance variations aligned with vertical gradients in acidity and ethanol content and were directly correlated with the synthesis of distinct volatile flavor compounds. Consequently, each layer developed unique flavor characteristics dominated by specific compounds, such as isoamyl acetate in the upper layer, phenolic compounds in the middle layer, and nonanal in the bottom layer. Furthermore, key microbial groups displayed significantly opposing patterns in their metabolic correlations. *Kroppenstedtia* and *Virgibacillus* showed positive correlations with a group of medium-chain esters but negative correlations with short-chain esters like ethyl acetate. Notably, *Pichia* and *Trichosporon* exhibited significantly opposite correlation patterns with multiple flavor compounds, potentially reflecting metabolic competition or niche complementarity that differentially shaped the flavor profile of each layer. These findings provided correlational insights into the ecological mechanism by which pit microorganisms collaboratively drove the formation of a complex flavor profile through spatial competition and metabolic complementarity. Future research will introduce a qPCR standard curve method to measure the absolute gene copy numbers per gram of fermented grain, thereby converting relative abundance into absolute abundance. This will allow for a more accurate assessment of microbial population dynamics and their contribution to flavor formation.

## Figures and Tables

**Table 1:** Alpha Diversity Index of Sample Bacterial and Fungal Communities.

Index	Bacteria			Fungi		
	JCU	JCM	JCB	JCU	JCM	JCB
ACE	38.08 ± 10.51 ^a^	85.14 ± 35.28 ^a^	162.53 ± 27.95 ^b^	72.57 ± 12.72 ^a^	94.05 ± 13.42 ^a^	84.00 ± 9.53 ^a^
Chao	36.04 ± 9.48 ^a^	84.47 ± 34.74 ^a^	162.96 ± 29.81 ^b^	72.33 ± 12.50 ^a^	94.00 ± 13.45 ^a^	84.00 ± 9.54 ^a^
Shannon	1.52 ± 0.26 ^a^	1.83 ± 0.57 ^a^	2.87 ± 0.60 ^b^	2.07 ± 0.71 ^ab^	3.09 ± 0.25 ^b^	1.68 ± 0.52 ^a^
Simpson	0.35 ± 0.13 ^a^	0.33 ± 0.21 ^a^	0.27 ± 0.16 ^a^	0.25 ± 0.17 ^a^	0.19 ± 0.27 ^a^	0.36 ± 0.16 ^a^
Coverage	0.99 ± 0.00 ^a^	0.99 ± 0.00 ^a^	0.99 ± 0.00 ^a^	0.99 ± 0.00 ^a^	0.99 ± 0.00 ^a^	1.00 ± 0.00 ^a^

Note: Different superscript letters above the bars indicate significant differences among groups (*p* < 0.05).

## Data Availability

The original contributions presented in this study are included in the article/Supplementary Materials. Further inquiries can be directed to the corresponding author.

## Supplementary Materials

The following supporting information can be downloaded at: https://www.mdpi.com/article/10.3390/foods15111849/s1. Figure S1. Reference for sampling sites of FG from the sauce-flavor baijiu pit. Table S1. Volatile compound content of samples based on GS-MS analysis. Table S2. Analysis of volatile compounds in fermented grains of different layers by ROAV.
