# Supplementary material for: Investigating the Spatial Heterogeneity of Microbial Communities and Flavor Compounds During the First Fermentation Stage of Sauce-Flavor Baijiu
Source: Foods. 2026 May 23;15(11):1849. doi: 10.3390/foods15111849 (PMC13256519; doi:10.3390/foods15111849)
Supplement: Supplementary file 1 [file foods-15-01849-s001.zip › foods-4240154-supplementary.pdf]

Supplementary information for

Investigating Spatial Heterogeneity in Microbial Communities and Flavor Compounds during the First Fermentation Stage of Sauce-Flavor Baijiu

Fangling Wen[1,2#], Feifei Lu[1#], Yu Zhao[1], Jiehua You[4], Shengfeng Li[4], MinnaYao[2], Xitao Cao[3], Yonghui Lin[1*]

[1] Fujian Key Laboratory of Quality and Safety of Agri-Products, Institute of Quality Standards and Testing Technology for Agro-products, Fujian Academy of Agricultural Sciences, Fuzhou 350003, China.

[2] College of Food Science, Fujian Agriculture and Forestry University, Fuzhou 350002, China.

[3] Jiangsu University of science and technology, Zhenjiang, Jiangsu 212100, China.

[4] Fujian Shuanglong Xizhu Baijiu Industry Co., Ltd., Jian'ou 353100, China.

* Correspondence:Yonghui Lin; E-mail: fjsnkylyh@163.com; Tel.: 13950262566;

[#] These authors contributed equally to this work.

S1. Experimental

S1.1 PCR conditions

Pre-denaturation at 95 °C for 3 min, followed by 30 cycles (denaturation at 95 °C for 30 sec, annealing at 55 °C for 30 sec, extension at 72 °C for 45 sec); final extension at 72 °C for 10 min, and storage at 4 °C. The 20 μL PCR mixture contained: 4 μL of 5× FastPfu buffer, 2 μL of 2.5 mM dNTPs, 0.8 μL each of forward and reverse primers (5 μM), 0.4 μL of FastPfu polymerase, 0.2 μL of BSA, 10 ng of DNA template, and added ddH_2O to volume.

S1.2 High Throughput Sequencing

PCR amplicons were recovered using 2% agarose gel electrophoresis, purified with a PCR clean up kit, eluted with elution buffer, and verified on a 2% agarose gel. Quantification was performed with a Synergy HTX microplate reader. Purified amplicons were sequenced on the Illumina MiSeq platform following the standard operating procedure to construct PE2×300 libraries.

S1.3 Formulas

The concentration of volatile odor compounds was calculated using the following formula:

$$C_i = (C_{is} \times A_i) / A_{is} \qquad (1)$$

Where: C_i and A_i represent the concentration and peak area of the volatile odor compound, respectively; C_{is} and A_{is} represent the concentration and peak area of the internal standard (2-octanol), respectively.

Relative Odor Activity Value (ROAV) Evaluation Method:

The ROAV method was used to evaluate the contribution of individual volatile components to the overall aroma of fermented grains. the component with the greatest contribution to the aroma of fermented grains was assigned a standard $ROAV_{stan}$ = 100. The ROAV for other volatile components was calculated using the following formula:

$$ROAV_i = (C_i/C_{stan}) \times (T_{stan}/T_i) \times 100 \qquad (2)$$

Where: C_i is the relative content of the volatile component; T_i is the odor threshold of the volatile component (μg/kg); C_{stan} is the relative content (%) of the component contributing the most to the aroma of fermented grains; T_{stan} is the odor threshold of the component contributing the most to the aroma of fermented grains (μg/kg).

S1.4 The detailed calculation formula in Table 1

The Chao index estimates the total number of undetected rare species in the community based on the numbers of species detected only once and twice.

$$S_{Chao1} = S_{obs} + \frac{n_{1(n_1-1)}}{2(n_2+1)}$$

Where: S_{chao1}: estimated number of ASVs; S_{obs}: observed number of ASVs; n_1: number of ASVs containing only one sequence (e.g., "singletons"); n_2: number of ASVs containing only two sequences (e.g., "doubletons").

The ACE index estimates the total number of species actually present in the community based on the number of rare species (abundance≤10) and their coefficient of variation.

$$S_{ACE} = \begin{cases} S_{abund} + \dfrac{S_{rare}}{C_{ACE}} + \dfrac{n_1}{C_{ACE}}\hat{\gamma}^2_{ACE}, & for\ \hat{\gamma}_{ACE} < 0.80 \\ S_{abund} + \dfrac{S_{rare}}{C_{ACE}} + \dfrac{n_1}{C_{ACE}}\tilde{\gamma}^2_{ACE}, & for\ \hat{\gamma}_{ACE} \geq 0.80 \end{cases}$$

$$N_{rare} = \sum_{i=1}^{abund} i\, n_i, C_{ACE} = 1 - \frac{n_1}{N_{rare}}$$

$$\tilde{\gamma}^2_{ACE} = \max\left[\frac{S_{rare}}{C_{ACE}}\frac{\sum_{i=1}^{abund} i(i-1)n_i}{N_{rare}(N_{rare}-1)} - 1{,}0\right]$$

$$\tilde{\gamma}^2_{ACE} = \max\left[\hat{\gamma}^2_{\mathrm{ACE}}\left\{1 + \frac{N_{rare}(1-C_{ACE})\sum_{i=1}^{abund} i(i-1)n_i}{N_{rare}(N_{rare}-C_{ACE})}\right\}, 0\right]$$

Where: n_1: number of ASVs containing one sequence; S_{rare}: number of ASVs containing "abund" sequences or fewer; S_{abund}: number of ASVs containing more than "abund" sequences; abund: threshold for "dominant" ASVs, default is 10.

The Simpson index calculates the probability that two randomly selected individuals belong to the same species (i.e., the sum of squares of relative abundances of each species), and subtracts this probability from 1 to represent diversity.

$$D_{Simpson} = \frac{\sum_{i=1}^{S_{obs}} n_i(n_i-1)}{N(N-1)}$$

Where: S_{obs}: number of observed ASVs; n_i: number of sequences contained in the i-th ASV; N: total number of all sequences.

The Shannon index is calculated as the negative sum of the product of the relative abundance of each species and its natural logarithm, comprehensively measuring both species richness and evenness.

$$H_{Shannon} = -\sum_{i=1}^{S_{obs}} \frac{n_i}{N}\ln\frac{n_i}{N}$$

Where: S_{obs}: number of observed ASVs; n_i: number of sequences contained in the i-th ASV.

The Coverage index calculates 1 minus the proportion of sequences belonging to species with only one sequence relative to the total number of sequences, evaluating how well the sequencing results cover the true condition of the sample.

$$C = 1 - \frac{n_1}{N}$$

Where: n_1: number of ASVs containing only one sequence; N: total number of sequences appearing in the sample.

S2. Supporting Date

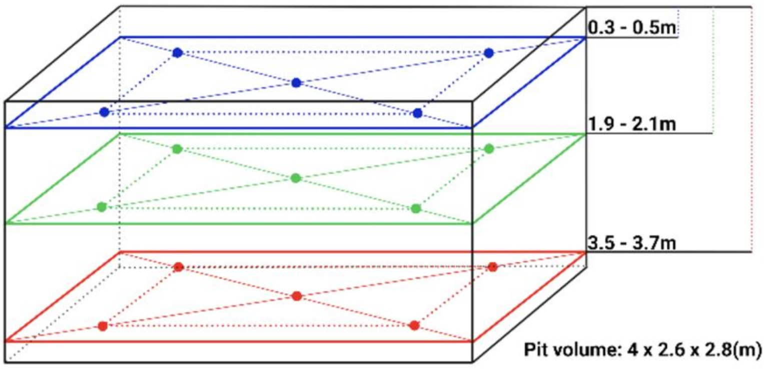

Figure S1 Reference for Sampling Sites of FG from the Sauce-flavor Baijiu Pit.

Table S1 Volatile compound content of samples based on GS-MS analysis

Compounds		Content (μg/kg）		
Number	Name	FGU	FGM	FGB
A1	Isoamyl alcohol	512.41±7.29[b]	608.20±9.43[a]	353.33±5.51[c]
A2	Isobutyl lactate	107.11±4.94[b]	181.46±5.02[a]	87.18±1.58[c]
A3	Ethyl acetate	158.46±6.16[a]	145.45±4.78[b]	120.22±3.56[c]
A4	Ethyl palmitate	130.08±5.77[b]	1.42±0.11[c]	144.70±2.65[a]
A5	Ethyl linoleate	50.66±2.01[b]	63.36±1.06[a]	43.54±0.88[c]
A6	Propyl acetate	91.34±5.05[b]	132.21±3.79[a]	71.15±3.72[c]
A7	Propyl palmitate	26.69±0.92[b]	39.66±1.93[a]	20.71±0.32[c]
A8	Ethyl phenylacetate	22.19±1.00[c]	32.62±1.22[a]	31.21±0.97[b]
A9	Ethyl oleate	31.03±1.94[b]	37.71±1.42[a]	27.64±0.89[c]
A10	Isoamyl acetate	57.64±2.93[a]	-	-
A11	Ethyl elaidate	3.11±0.25[c]	21.58±0.72[b]	21.65±0.34[a]
A12	DL-2-hydroxy-4-methylvalerate ethyl ester	9.70±0.84[c]	19.77±0.77[a]	16.47±0.56[b]
A13	Ethyl octanoate	6.35±0.60[c]	10.25±0.69[b]	13.46±0.32[a]
A14	4-Hydroxy-3-methyl-2-butanone	77.19±0.73[a]	-	-
A15	1-Pentadecene	6.72±0.43[b]	9.11±0.39[a]	9.11±0.12[a]
A16	Diisobutyl methanol	4.15±0.36[c]	10.42±0.62[a]	5.46±0.28[b]
A17	Ethyl phenylacetate	4.44±0.38[c]	7.35±0.41[b]	8.22±0.20[a]

A18	Ethyl laurate	5.11±0.39^{c}	7.05±0.44^{a}	6.74±0.22^{b}
A19	Propyl hexanoate	2.68±0.15^{c}	8.80±0.36^{a}	5.48±0.10^{b}
A20	2-Methoxy-4-methylphenol	4.87±0.31^{b}	11.52±1.19^{a}	3.56±0.09^{c}
A21	Ethyl decanoate	3.47±0.35^{c}	5.06±0.47^{b}	6.91±0.30^{a}
A22	Propyl caprylate	2.85±0.22^{c}	5.27±0.15^{a}	4.44±0.14^{b}
A23	Isopentyl lactate	-	12.33±0.42^{a}	-
A24	Ethyl hexadecenoate	2.45±0.70^{c}	5.51±0.20^{a}	4.33±0.07^{b}
A25	n-Hexadecane	1.03±0.17^{c}	7.15±0.61^{b}	8.71±0.13^{a}
A26	Nonanal	-	-	8.39±0.31^{a}
A27	Ethyl nonanoate	1.54±0.19^{c}	2.44±0.21^{b}	3.38±0.07^{a}
A28	Ethanol	2.64±0.30^{b}	3.93±0.11^{a}	1.68±0.04^{c}
A29	n-Dodecane	-	4.07±0.33^{b}	4.45±0.13^{a}
A30	Propanoic acid-2-phenylethyl ester	2.15±0.15^{b}	3.55±0.25^{a}	2.13±0.09^{c}
A31	n-Propyl decanoate	1.33±0.11^{c}	2.07±0.09^{a}	1.63±0.04^{b}
A32	Ethyl stearate	1.92±0.31^{b}	1.98±0.16^{a}	1.81±0.03^{c}
A33	Propyl myristate	1.35±0.09^{b}	2.11±0.10^{a}	1.05±0.04^{c}
A34	Propyl laurate	1.14±0.07^{b}	1.90±0.10^{a}	1.02±0.03^{c}
A35	Diethyl succinate	0.58±0.08^{c}	2.09±0.11^{b}	2.15±0.05^{a}
A36	2-Bromo-dodecane	-	-	2.61±0.07^{a}
A37	4-Ethyl-2-methoxyphenol	-	3.00±0.25^{a}	-
A38	Tetradecane	-	2.47±0.11^{a}	-
A39	n-Octadecane	-	1.26±0.07^{a}	0.90±0.02^{b}
A40	n-Heptadecane	0.71±0.07^{c}	1.72±0.08^{b}	2.12±0.07^{a}

A41	Ethyl heptadecanoate	0.85±0.09[b]	-	1.62±0.05[a]
A42	Chloromethyl nonyl ester	1.77±0.09[a]	-	-
A43	3,8-Dimethyldecane	-	1.35±0.08[a]	-
A44	7-Methylheptadecane	-	-	0.77±0.03[a]

Note: Different lowercase letters (a, b, c) indicate that the same compound has significant differences between different cellar entry times, $P<0.05$. (-: not detected).

TableS2 Analysis of volatile compounds in fermented grains of different layers by ROAV.

Number	Name	CAS	Odor threshold	ROAV		
				FGU	FGM	FGB
A1	Isoamyl alcohol	123-51-3	5000	9.2368	7.4580	4.5284
A2	Isobutyl lactate	585-24-0	1200	8.0449	9.2714	4.6556
A3	Ethyl acetate	141-78-6	1000	14.2821	8.9178	7.7039
A4	Ethyl palmitate	628-97-7	16000	0.7328	0.0054	0.5795
A5	Ethyl linoleate	544-35-4	15000	0.3044	0.2590	0.1860
A6	Propyl acetate	109-60-4	1500	5.4884	5.4040	3.0396
A7	Propyl palmitate	2239-78-3	15000	0.1604	0.1621	0.0885
A8	Ethylphenylacetate	103-45-7	20	100.0000	100.0000	100.0000
A9	Ethyl oleate	111-62-6	12000	0.2331	0.1927	0.1476
A10	Isoamyl acetate	19329-89-6	200	25.9757	0.0000	0.0000
A11	Ethyl elaidate	6114-18-7	13000	0.0216	0.1018	0.1067
A12	DL-2-hydroxy-4-methylvalerate ethyl ester	10348-47-7	500	1.7485	2.4243	2.1109
A13	Ethyl octanoate	106-32-1	80	7.1541	7.8556	10.7818
A14	4-Hydroxy-3-methyl-2- butanone	3393-64-4	100	69.5719	0.0000	0.0000
A15	1-Pentadecene	13360-61-7	200000	0.0030	0.0028	0.0029
A16	Diisobutyl methanol	108-82-7	30000	0.0125	0.0213	0.0117
A17	Ethyl phenylacetate	101-97-3	15	26.6787	30.0429	35.1169
A18	Ethyl laurate	106-33-2	5000	0.0921	0.0865	0.0864
A19	Propyl hexanoate	626-77-7	60	4.0258	8.9924	5.8528
A20	2-Methoxy-4-methylphenol	93-51-6	20	21.9468	35.3158	11.4066
A21	Ethyl decanoate	110-38-3	1200	0.2606	0.2585	0.3690
A22	Propyl caprylate	624-13-5	100	2.5687	3.2311	2.8452
A23	Isopentyl lactate	19329-89-6	800	0.0000	0.9450	0.0000
A24	Ethyl hexadecenoate	54546-22-4	800	0.2760	0.4223	0.3468

A25	n-Hexadecane	544-76-3	500000	0.0002	0.0009	0.0011
A26	Nonanal	124-19-6	5	0.0000	0.0000	107.5296
A27	Ethyl nonanoate	123-29-5	150	0.9253	0.9973	1.4440
A28	Ethanol	64-17-5	10000000	0.0000	0.0000	0.0000
A29	n-Dodecane	112-40-3	100000	0.0000	0.0025	0.0029
A30	Propanoic acid-2-phenylethyl ester	122-70-3	8	24.2226	27.2072	17.0618
A31	n-Propyl decanoate	30673-60-0	1500	0.0799	0.0846	0.0696
A32	Ethyl stearate	111-61-5	20000	0.0087	0.0061	0.0058
A33	Propyl myristate	14303-70-9	10000	0.0122	0.0129	0.0067
A34	Propyl laurate	3681-78-5	6000	0.0171	0.0194	0.0109
A35	Diethyl succinate	123-25-1	2500	0.0209	0.0513	0.0551
A36	2-Bromo-dodecane	13187-99-0	50000	0.0000	0.0000	0.0033
A37	4-Ethyl-2-methoxyphenol	2785-89-9	50	0.0000	3.6787	0.0000
A38	Tetradecane	629-59-4	300000	0.0000	0.0005	0.0000
A39	n-Octadecane	593-45-3	800000	0.0000	0.0001	0.0001
A40	n-Heptadecane	629-78-7	400000	0.0002	0.0003	0.0003
A41	Ethyl heptadecanoate	14010-23-2	18000	0.0043	0.0000	0.0058
A42	Chloromethyl nonyl ester	57045-82-6	10000	0.0160	0.0000	0.0000
A43	3,8-Dimethyldecane	17312-55-9	150000	0.0000	0.0006	0.0000
A44	7-Methylheptadecane	20959-33-5	300000	0.0000	0.0000	0.0002

Note: ROAV≥1: the substance contributes to the overall aroma; ROAV < 1: this substance has a weak contribution to the current aroma.
